# Choline Kinase, A Novel Drug Target for the Inhibition of *Streptococcus pneumoniae*

**DOI:** 10.3390/antibiotics6040020

**Published:** 2017-09-25

**Authors:** Tahl Zimmerman, Salam Ibrahim

**Affiliations:** Food Microbiology and Biotechnology Laboratory, Department of Family and Consumer Sciences, College of Agriculture and Environmental Sciences, North Carolina Agricultural and Technical University, 1601 East Market Street, Greensboro, NC 27411, USA; ibrah001@ncat.edu

**Keywords:** *Streptococcus pneumonia*, choline kinase, Gram-positive, lipoteichoic acid, cell wall, hemocholinium-3

## Abstract

Gram-positive pathogens, such as *Streptococcus pneumoniae*, can have deleterious effects on both human and animal health. Antibiotics and antimicrobials have been developed to treat infections caused by such pathogens and to prevent food contamination. However, these strategies have been increasingly thwarted by the emergence of resistant bacteria strains. Thus, new methods for controlling Gram-positive pathogen growth need to be continuously developed. Choline analogs, such as Hemicholinium-3 (HC-3), have been shown to be useful in blocking cell division in eukaryotic cells through the inhibition of choline kinase, an enzyme which catalyzes the production of phosphocholine from choline and ATP. In some Gram-positive pathogens, choline kinase is an important enzyme in the production of the cell wall element, lipoteichoic acid. However, it is not known if inhibiting this enzyme has any effect on cell division in Gram-positive bacteria. Using the R6 strain as a model, we tested the ability of HC-3 to block the activity of choline kinase in *S. pneumoniae* and inhibit cell growth. Mass-spectrometry measurements of crude extracts revealed that HC-3 blocked choline kinase activity. Turbidity measurements and population counts showed that HC-3 inhibited cell growth. Competition assays with choline suggested that HC-3 also blocked choline transporters. Western blots showed that lipoteichoic acid production was blocked in the presence of HC-3, and autolytic assays showed that this decrease in lipoteichoic acids caused cells to be more resistant to autolysis. Scanning electron microscopy revealed that HC-3 distorted the cell wall. This study thus establishes choline kinase as a novel drug target for *S. pneumoniae*.

## 1. Introduction

From the nosocomial *Streptococcus pneumoniae* to the food-borne pathogen *Baccilus cereus*, Gram-positive pathogens are known to have deleterious effects on human and animal health. Efforts to treat infections or prevent food contamination are often complicated by the emergence of strains resistant to current treatments. For this reason, it is important to discover new methods for eliminating pathogens, which includes the development of novel drug targets. In order to take advantage of the current knowledge base, a useful strategy for discovering new drug targets in prokaryotic pathogens such as *S. pneumoniae* would be to determine whether enzymatic pathways that have, in the past, been modulated successfully in eukaryotic systems to treat diseases can be likewise exploited to prevent the growth of bacterial pathogens.

Choline kinase is a well-established drug target in eukaryotic systems [1]. Many inhibitors have been developed that are known to inhibit human choline kinase (hChok). These inhibitors have been shown to block the proliferation of cancer cells [2], to destroy parasites such as *Plasmodium falciparum* [3], and to treat autoimmune diseases [4]. *S. pneumoniae* is a pathogen known to express choline kinase [5]. Other Gram-positive pathogens, such as *Staphylococcus aureus, Bacillus subtilis, Clostridium perfringens*, and *Clostridium botulinum*, carry a putative choline kinase gene. It is unknown if blocking the activity of Gram-positive choline kinases is a viable method for blocking the growth of pathogens carrying the choline kinase gene.

The crystal structure of *S. pneumoniae* choline kinase (sChok) has been resolved, and its three-dimensional structure has been found to be homologous to the human variant of choline kinase [5]. This observation suggests that inhibitors that used to inhibit hChok could also inhibit sChok.

**Figure 1:**
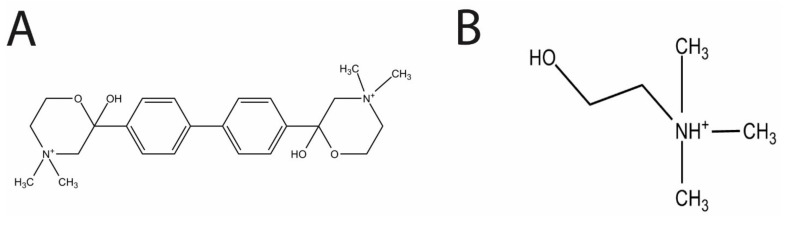
The respective structures of Hemicholinium-3 (HC-3) (**A**) and choline (**B**). The two methyl groups of HC-3 mimic the methyl groups found in choline.

Choline is a known essential nutrient for *S. pneumoniae* [6]. Choline (Figure 1B) is also a precursor molecule involved in the production of the two types of teichoic acid, lipoteichoic acid (LTA) and cell wall teichoic acid (CTA) [7,8]. In *S.pneumoniae*, both acids consist of the same types of polysaccharides. LTAs are attached to a lipid and embedded in the cell membrane, and CTAs are attached to the cell wall peptidoglycan layer in the cell wall [9]. Lipoteichoic acid is an important virulence factor whose production is a known drug target [10]. In *Staphylococcus aureus*, the teichoic acid synthesis pathway has been validated as a possible drug target, and has been shown to mediate resistance to Beta-lactam drugs [11].

In prokaryotes such as *S. pneumoniae*, choline kinase is part of the CDP-choline pathway whose final product is teichoic acid. The gene operon *lic* is responsible for the CDP-choline pathway in *S. pneumoniae* that ultimately participates in the production of teichoic acid. The choline transporter, LicB, acquires choline from the external environment. Choline is phosphorylated by the choline kinase LicA to produce phosphocholine. Phosphocholine is then converted into CDP-choline by cytidylyl transferease LicC. LicD1 and LicD2 phosphocholine transferase transfer the phosphocholine in CDP-choline to pre-teichoic acid glycan to make teichoic acid [12]. Teichoic acid is then transferred across the cell membrane by teichoic acid flippase TacF for integration into the cell membrane and wall. A LidD2 knockout in *S. pneumoniae* was shown to reduce the amount of teichoic acid that contained choline phosphate. This mutated strain exhibited reduced virulence in mouse models and reduced nasopharyngeal colonization as well as reduced adherence to alveolar cells [11]. Incorporation of choline into the cell wall is important for proper cell functioning, because this molecule acts as an anchor for choline-binding proteins (CBP), such as members of the murein hydrolase family [13]. Murein hydrolases help remodel the cell wall during bacterial growth and cell division.

In light of the fact that teichoic acids are important components of the cell wall, and that the choline kinase enzyme lies in the metabolic pathway that produces teichoic acids, it is likely that: (1) inhibitors designed to block the activity of choline kinase in eukaryotic cells will also block the choline kinase expressing *S. pneumoniae*; and, (2) inhibiting choline kinase in in *S. pneumoniae* will inhibit the growth of this pathogen.

It is imperative that new drug targets be discovered in *S. pneumoniae* and other pathogens as older antibiotics become obsolete through the evolution of resistant strains. Drugs that are used to prevent diseases involving eukaryotic cells could be repurposed as antibiotics and antimicrobials for the control of Gram-positive pathogens. The objective of this study was to evaluate choline kinase as a possible drug target for *S. pneumoniae* using the R6 strain as a model.

In the present study, we evaluated the effects of the choline analog and known inhibitor of eukaryotic choline kinase, hemicholinium-3 (HC-3, Figure 1A), on *S. pneumoniae* choline kinase activity and cell growth.

HC-3 was chosen as a test inhibitor because it is a known inhibitor of both cancerous [14] and parasite cells [3]. The crystal structure in complex with human choline kinase was solved, and was shown to enter the choline binding site, demonstrating that HC-3 was competitive with the choline substrate [15]. In addition, HC-3 was shown to block choline transporters in eukaryotic cells [16].

## 2. Results

### 2.1. Hemocholinium-3 Inhibits Choline Kinase Activity

**Figure 2:**
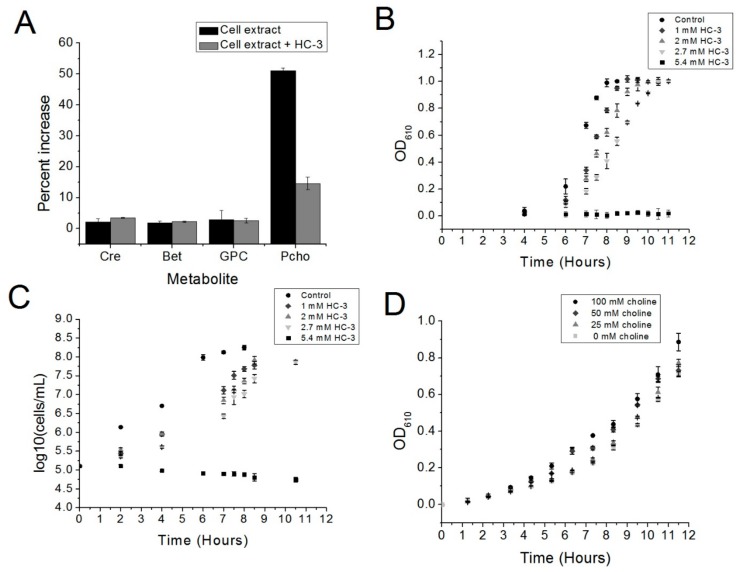
(**A**) The ability of HC-3 to inhibit the choline kinase enzymatic activity of *S. pneumoniae* cell extracts was monitored by mass spectrometry. The concentrations of glycerophosphocholine (GPC), betaine (BET), and creatinine (Cre) were quantified as a control for the amount of cell extract in each reaction. Reactions were normalized against a control reaction lacking ATP (not seen). There was a notable increase in the amount of phosphocholine in solution when ATP was added to the reaction mix (Cell extract alone), and an evident reversal of phosphocholine production when HC-3 was added (Cell extract + HC-3). (**B,C**) R6 cells were grown in either a BHI-CAT control or increasing concentrations of Hemicholinium-3 (HC-3). (**B**) Cell growth was monitored by measuring the optical densities (O.D.) of the cultures at 610 nm. (**C**) R6 cell viability was determined by measuring the bacterial populations using plating methods. (**D**) R6 cells were grown in the presence of a fixed amount of HC-3 (2.7 mm) and varying concentrations of choline. Cell growth was monitored by measuring the optical density at 610 nm.

The incubation of cell extract with choline and ATP led to the production of phosphocholine, as measured by LC/MS. Fifty percent more phosphocholine was detected relative to a “no-ATP” control after one-hour incubation of the reaction mixture at 37 °C (Figure 2A). Phosphocholine production in the presence of choline and ATP was expected, since R6 is known to express choline kinase (Figure 2A) [5]. Adding HC-3 substantially reduced the production of phosphocholine relative to the “no-ATP” control (Figure 2A), which correlates with the known activity of HC-3 and other choline analogs against eukaryotic choline kinase homologs. This result indicated that HC-3 could be blocking choline kinase activity.

### 2.2. Hemicholinium-3 Inhibits R6 Cell Growth

In order to determine if choline kinase might be a target in *S. pneumoniae*, the choline analog Hemicholinium-3 (HC-3) was added to cultures of R6 cells to determine the effect of HC-3 on cell growth. HC-3 delayed the growth of the R6 strain of *S. pneumoniae* in a dose-dependent fashion. This result is reflected in both turbidity measurements (Figure 2B) and measurements of bacterial populations (Figure 2C). Meanwhile, growth was completely inhibited at a HC-3 concentration of 5.4 mM, as determined by Optical Density (OD) measurements.

### 2.3. Excess Choline Can Moderately Reverse the Effects of HC-3

To determine whether or not HC-3 acted in a manner competitive with the natural substrate choline, increasing amounts of choline were added to cultures containing 2.7 mM of HC-3 (The amount of choline could not be increased beyond 100 mM, at which point the choline itself became inhibitory (data not shown). Choline somewhat reversed the effects of HC-3 in a dose-dependent manner (Figure 2D). The competition between HC-3 and choline suggested that HC-3 may be blocking choline transporters, as has been shown in eukaryotic systems [17].

### 2.4. HC-3 Reduces the Amount of Lipoteichoic Acid in Cell Walls

**Figure 3:**
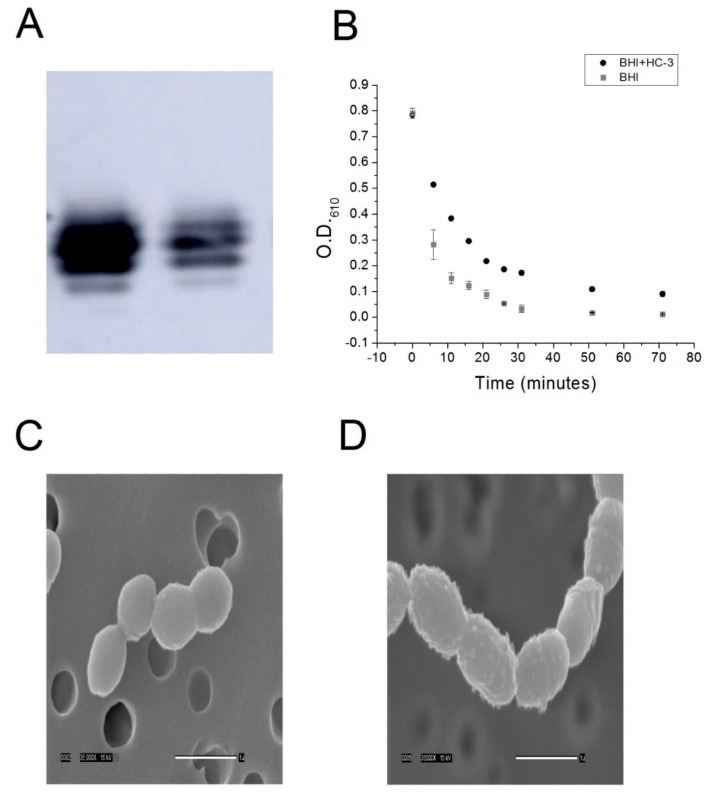
(**A**) Western blot detection of lipoteichoic acid from cell extracts of either control R6 cells (left lane) or R6 cells cultured in the presence of 2.7 mM HC-3 (right lane).The intensity of the signal drops dramatically (2.4×) in the case of treatment with HC-3. (**B)** Cell lysis of cells that had been grown in either BHI alone or BHI + HC-3 were then exposed to a deoxycholate-containing solution. Cell lysis was monitored by measuring the optical density of the solution at 610 nm. (**C,D**) Scanning electron micrographs at 35,000× magnification of untreated *S. pneumoniae* cells and (**C**) and cells treated with HC-3 (**D**). The smooth cell surface changes into a bumpy surface upon treatment with HC-3, indicating that the cell wall structure has been affected.

In order to determine if HC-3 was blocking the activity of choline kinase, the production of lipoteichoic acid was monitored by Western blot. Lipoteichoic acid is a metabolite that is downstream from a biochemical pathway of which choline kinase is a part [8]. Choline kinase phosphorylation of choline to produce phosphocholine is an important step in the pathway that leads to choline incorporation into lipoteichoic acid. As such, lipoteichoic acid contains phosphocholine moieties and can be detected using an anti-phosphocholine antibody [18]. By detecting the amount of phosphocholine in the cell wall, we could infer differences in the amount lipoteichoic acid embedded in the cell wall. Inhibiting choline kinase would be expected to reduce the production of lipoteichoic acid. Consistent with this model, HC-3 reduced the amount of lipoteichoic acid present in the cell wall, as determined by Western blot (Figure 3A).

### 2.5. Cells Grown in the Presence of HC-3 Are More Resistant to Autolysis

In order to confirm the Western blot result, R6 cells were grown to saturation in the presence or absence of HC-3. If the production of lipoteichoic acid was reduced on the cell wall as a consequence of treatment with HC-3, one would expect cells treated with HC-3 to be more resistant to autolysis. Lipoteichoic acid is the component of the cell wall that anchors the autolytic protein autolysin in the first step of a two-step process which leads to autolysin cleavage of peptidoglycan and subsequent cell lysis [19]. By reducing the production of lipoteichoic acid, fewer molecules of autolysin can bind to the cell surface. Fewer docking sites for autolysin should translate into a slower rate of autolysis. To test this idea, control and treated cells were exposed to a deoxycholate/phosphate solution. Phosphate is known to induce autolysis in Gram-positive bacterial cells [19], and deoxycholate, a detergent, has been known to induce the release of autolysin into solution, allowing the autolysin to interact with the cell wall, leading to cell lysis [20]. Cells grown in the presence of HC-3 were more resistant to autolysis induced by this detergent mixture with respect to a non-autolytic control (see Figure 3B). This confirmed a model whereby inhibition of choline kinase and choline transporters led to a reduction in the concentration of lipoteichoic acid in the cell wall.

### 2.6. HC-3 Distorts the Structure of Cell Walls

If the concentration of lipoteichoic acid in the cell wall of a Gram-positive cell such as *S. pneumoniae* were reduced, we would also expect the cell wall to be distorted in some way. This is because lipoteichoic acid plays an important role in the structure of the cell wall. In order to determine if HC-3 caused any distortions in the cell wall, Scanning Electron Microscopy was performed on control cells and on cells grown in the presence of HC-3. As shown in Figure 3C,D, the surface of the cell wall changed from very smooth to very bumpy, indicating that the cell was affected in the presence of HC-3. HC-3 inhibited choline kinase, which blocked the production of phosphocholine. This reduced the production of lipoteichoic acid, resulting in distortions in the cell walls and cell resistance to lysis. In addition, the cells were elongated, indicating that the cell division process was affected, which would be expected if the autolytic machinery were being modulated.

## 3. Discussion

Our preliminary data have shown that choline kinase is a promising new drug target in *S. pneumoniae*. The choline analog HC-3 inhibited the growth of *S. pneumoniae* and blocked the activity of choline kinase in crude extracts, suggesting that using choline analogs to inhibit the growth of R6 cells is a promising strategy and that choline kinase inhibitors, such as HC-3, have the potential to be employed against virulent strains of *S. pneumoniae*.

HC-3 is a known inhibitor of choline kinase in eukaryotic systems. Phosphocholine is a key element of many cell membrane components in eukaryotic cells, and interfering with its production in the cell membrane affects cell division. Therefore, reducing the production of phosphocholine is an established strategy for blocking the proliferation of cancer cells and cell division in parasites [1], and a number of drugs that inhibit choline kinase have been developed for that purpose [1]. The results presented here suggest that choline kinase inhibitors could also be developed as antibiotics against *S. pneumoniae*.

However, the mechanism of action of any choline analog could simultaneously inhibit the action of bacterial choline-binding proteins (CBP) other than choline kinase. For example, bicyclic amine choline analogs have been shown to be effective in bacterial systems against CBPs [13]. Nevertheless, choline kinase is a promising new CBP drug target for *S. pneumoniae.*

Further studies on the purified enzyme are warranted in order to determine if the mechanism of action of HC-3 is the same on an enzymatic level as it is for *S. pneumoniae* choline kinase. In addition, we intend to explore whether this system could be used to block the growth of virulent strains of *S. pneumoniae* as well other Gram-positive pathogens, such as *B. cereus* and *S. aureus*. We would also want to investigate whether other, more potent, inhibitors of human choline kinase could be repurposed to block the growth of choline-kinase-carrying Gram-positive pathogens more effectively than has been shown for HC-3. HC-3 is known to be a weak inhibitor in eukaryotic systems, and has been shown to be weak in the present investigation as well.

Once more potent inhibitors have been identified, the effect of choline kinase inhibitors on the invasiveness of *S. pneumoniae* and other Gram-positive pathogens should be explored. In addition, we intend to assess the effects of choline kinase inhibitors on other systems, such as *Lactococcus lactis* and *Lactobacillus cremoris*, whose autolytic activity must be modulated during cheese production. Growing these strains in the presence of choline kinase inhibitors is also a promising strategy for modulating autolytic activity by repressing the production of lipoteichoic acid.

In summary, this work establishes choline kinase as a novel drug target in *S. pneumoniae* and has opened the door to further study into applications for the modulation of choline kinase activity in *S. pneumoniae* and other Gram-positive bacteria.

## 4. Methods and Materials

### 4.1. Cell Culture

Five μL of a glycerol stock of the *S. pneumoniae* R6 strain was used to inoculate 5 mL of Brain Heart Infusion broth (BHI, Accumedia, Sydney, Australia) supplemented with 5 units/mL catalase (BHI-CAT). This starter culture was incubated at 37 °C in a water bath until an optical density at 610 nm (OD_610_) was reached, as measured by a spectrophotometer (Evolution 201, GE Healthcare, Wauwatosa, WI, USA). Forty μL of this starter culture was used to inoculate 40 mL of BHI-CAT media with or without varying concentrations of HC-3. This culture was then incubated at 37 °C in a Precision water bath (Thermo Scientific, Walthum, MA, USA).

### 4.2. Mass Spectrometry Quantification of Phosphocholine

An *S. pneumoniae* R6 cell culture in 5 mL BHI-CAT was grown to an OD_610_ of 1.0 and then spun down, washed three times in 100 mM Tris pH 8, and then resuspended in 250 μL in 100 mM Tris pH 8. Glasperlin 0.1 mm diameter glass beads (Sartorius Stedim, Göttingen, Germany) were added, and the cells were lysed using a bead beater. Thirty-six μL of R1 buffer (333 mM, Tris, pH 8, 33 mM MgCl_2_, 3.3 mM choline) or R2 buffer (R1 buffer + 2.7 mM Hemicholinium-3) was added to 72 μL of cell extract. Twelve μL of either 100 mM ATP or water were then added to the reaction mixtures. A total of three reactions were prepared (“−ATP”, “+ATP”, and “+ATP + HC-3”) and incubated for 37 °C in a water bath, followed by inactivation at 95 °C for 5 min. Denatured protein was removed by centrifugation at 11,000× *g* followed by pipetting of the supernatant into a fresh tube.

Samples were spiked with stably labeled internal standards for each analyte and extracted using a modified reported method [21]. Next, the samples were extracted with methanol/chloroform (2:1, *v*/*v*), vortexed, and incubated at −20 °C overnight. Samples were then centrifuged, supernatants retained, and the pellets were re-extracted with methanol/chloroform/water (2:1:0.8, *v*/*v*/*v*). After vortexing and centrifugation, the supernatants were combined with the original supernatants. Water and chloroform were then added to the supernatants to induce phase separation. After centrifugation, the upper aqueous phase containing choline, phosphocholine, glycerophosphocholine, betaine, and creatinine was ready for analysis using liquid chromatography-stable isotope dilution-multiple reaction monitoring mass spectrometry (LC-SID-MRM/MS).

Chromatographic separations were performed on an Acquity UPLC HILIC 1.7 µm 2.1 × 50 mm column (Waters Corp, Milford, MA, USA) using a Waters UPLC system. Column temperature was 40 °C, and the flow rate was 0.37 mL/min. The mobile phases were: A: 0.125% formic acid in water, and B: 90% acetonitrile/10% water, 10 mM ammonium formate, and 0.125% formic acid. For aqueous analytes, the gradient was 0% A/100% B to 0.1 min, 30% A to 2.5 min, 62.5% A to 3.5 min, and 0% A to 4 min. The analytes and their corresponding isotopes were monitored on a Waters TQ detector using characteristic precursor–product ion transitions. Concentrations of each analyte in the samples were determined using the peak area ratio of the analyte to its isotope.

### 4.3. Monitoring Cell Growth and Viability

Cell growth was monitored over time by measuring the turbidity of the cultures at OD_610_. Cell viability was monitored by plating aliquots of each culture on Mueller Hinton Agar plates with 5% sheep’s blood at several intervals. Both cell growth and cell viability were monitored until the cultures reached saturation (reaching an OD_610_ of about 1.0).

### 4.4. Choline/HC-3 Competition Assay

R6 cells were cultured as described above, except that in all conditions, the AGCH minimal media contained 2.7 mM of HC-3 (BHI-CAT-H). Increasing concentrations of choline were added to the BHI-CAT-H. Cultures were inoculated with 0.2% of a starter culture previously grown to an OD_610_ of 0.5, and cell growth was monitored by measuring OD_610_ using a spectrophotometer until the cultures reached saturation.

### 4.5. Lipoteichoic Acid Detection by Western Blot

R6 cells were cultured to saturation in either BHI-cat alone or BHI-cat + 2.7 mM HC-3. One milliliter (1 mL) of cell samples was centrifuged at 3000 *g*, resuspended in 50 μL 6 M urea, and then incubated at 37 °C for 5 min to solubilize the protein. Protein quantification was performed using the BCA method (BioRad, Hercules, CA, USA), and 2.3 ng protein samples were incubated with a Quickstain solution (GE Healthcare, Wauwatosa, WI, USA). The samples were then loaded onto a 16% SDS-PAGE gel and transferred onto a low fluorescence PVDF membrane (GE Healthcare). Next, the membrane was incubated with a 1:5000 anti-phosphocholine monoclonal antibody (SSI Diagnostica, Hillerød, Denmark ), followed by incubation with a 1:3000 secondary, anti-mouse antibody (Santa Cruz Biotech, Santa Cruz, CA, USA). Chemoluminescence was induced with ECL Prime (GE Healthcare) and detected using the Amersham Imager 600 (GE Healthcare).

### 4.6. Cell Lysis Assay

R6 strain *S. pneumoniae* cells were cultured to saturation in either an R1 buffer or R2 buffer. Five milliliter (5 mL) aliquots of cells were harvested by centrifugation, washed three times in a P buffer (12.5 mM phosphate, pH 8), and then resuspended in a P buffer + 0.125% deoxycholate. Cell lysis was monitored by measuring the optical density of the resuspension at 610 nm using a spectrophotometer.

### 4.7. Scanning Electron Microscopy

R6 was cultured to saturation in 50 mL of either BHI-cat media or BHI-cat containing 2.7 mm HC-3. Tubes containing bacteria were spun for 10 min at 2500 rpm and the supernatant removed. Next, 10 mL of 3% GTA in 0.1 M Na cacodylate buffer pH 7.4 were added to each tube, the pellet was resuspended, and the tubes were stored at 4 °C until processed. To prepare the bacteria for SEM processing, two 10 mL syringes were fitted with 13 mm Swinney filter holders fitted with a 0.4 µm Nucleopore filter. Three milliliters (3 mL) of buffer were placed in each syringe, to which 10 drops of resuspended bacteria in fix were added and gently filtered through. The filters were removed from the holders and placed sample side down in a vial containing fresh buffer. The filters were washed through three 30 min changes of buffer and then dehydrated with 30 min changes of cold 30%, 50%, 70%, and 95% ethanol. The first 30 min change of 100% ethanol was started cold, and the samples were warmed to room temperature (RT). Dehydration was completed with two 30 min changes of room temperature 100% ethanol. The samples were then critical point dried (Tousimis Samdri-795, Tousimis Research Corp, Rockville, MD, USA) in liquid CO_2_ and held for 10 min at critical point. Filters were mounted on stubs with double-stick tape and silver paint and then placed in a vacuum desiccator until coated. Samples were sputter-coated (Hummer 6.2 sputtering system, Anatech, Union City, CA, USA) with 50 Å Au/Pd and then viewed using a JEOL JSM-5900LV SEM (JEOL, Peabody, MA, USA). Images were acquired at a resolution of 1280 × 960 pixels.

## 5. Conclusions

Repurposing drugs that are used to prevent diseases involving eukaryotic cells for use as antibiotics and antimicrobials is a promising drug discovery strategy. The present research has demonstrated that choline kinase inhibitors could potentially be used as antibiotics for the control of Gram-positive pathogens. We present data here showing that the known eukaryotic inhibitor and choline analog, Hemicholinium-3, is effective in slowing the growth of the Gram-positive bacteria *S. pneumoniae* via inhibition of choline kinase. This study thus establishes a choline kinase as a novel target for *S. pneumoniae*. Inhibiting choline kinase is a method that may also be applicable to other Gram-positive pathogens that express choline kinase, which may have diverse applications in human health and food safety.
